# Single Passage of Human Metapneumovirus in LLC-MK2 Cells Does Not Affect Viral Protein-Coding Capacity

**DOI:** 10.1128/genomeA.00440-18

**Published:** 2018-05-24

**Authors:** Simon Loevenich, Aleksandr Ianevski, Eneli Oitmaa, Denis E. Kainov, Marit W. Anthonsen

**Affiliations:** a Department of Clinical and Molecular Medicine, Norwegian University of Science and Technology, Trondheim, Norway; b Institute of Genomics, University of Tartu, Tartu, Estonia; c Institute of Technology, University of Tartu, Tartu, Estonia

## Abstract

Here, we report the complete genome sequences of human metapneumovirus (HMPV) prior to and after passaging in LLC-MK2 cells. Paired comparisons of the 13,335-nucleotide genomes revealed that the virus acquired the T10736C transition in its genome, which did not affect the amino acid sequences of HMPV proteins.

## GENOME ANNOUNCEMENT

Human metapneumovirus (HMPV) is a negative-sense single-stranded RNA virus of the family Pneumoviridae. The virus may cause severe lower respiratory tract infections in young children (1). HMPV is also an important cause of disease in older adults (1). HMPV is often propagated in cells for serological assays and genetic tests, as well as for *in vitro* research purposes. It was shown that serial passaging of the HMPV A2 NL/00/17 strain, which is similar to the Canadian clinical isolate CAN97-83 (2), resulted in frequent frameshift and point mutations in the SH gene in LLC-MK2 cells (3). However, it is unknown if a single passage of this HMPV strain in LLC-MK2 cells could lead to an accumulation of mutations in the viral genome and affect its virus protein-coding capacity.

To answer this question, we sequenced HMPV genomes prior to and after passaging in LLC-MK2 cells. In particular, we infected LLC-MK2 cells with the HMPV A2 strain, which was passaged 3 times in the cells prior to the experiment (P3), at a multiplicity of infection of 0.01. After 8 days of cultivation, we harvested the virus (P4). We purified P3 and P4 by centrifugation on a 20% sucrose cushion. We isolated viral RNA from P3 and P4 samples using an RNeasy Plus minikit (Qiagen). The RNA was prepared for sequencing using a TruSeq stranded total RNA LT kit with Ribo-Zero Gold. Sequencing was done using an Illumina HiSeq 2500 instrument (setup, SR 1 × 50 bp + single index; sequencing kit, HiSeq Rapid SR cluster kit version 2; flow cell version, RapidRunV2, 300 million reads per flow cell; RTA version 1.18.64). Reads were aligned using the Bowtie 2 software package version 2.3.4.1 to the reference HMPV A2 NL/00/17 genome (GenBank accession number FJ168779). The preprocessing of alignments was done using Picard toolkit version 2.18.1. SAMtools version 1.x, and BCFtools were used for variant calling.

We found that upon propagation, HMPV acquired one point mutation in the viral genome. A T-to-C transition was found at position 10736 in P4. This variant resides in the open reading frame (ORF) of the L gene (nucleotides [nt] 7134 to 13151) and does not affect the amino acid sequence of viral RNA-dependent RNA polymerase (RdRP). Thus, this variant cannot confound serological assays commonly used in HMPV research or interfere with antiviral drug and vaccine development where virus propagation in cell culture is needed.

### Accession number(s).

Two complete genome sequences of HMPV NL/00/17 type A2 have been deposited in GenBank under accession numbers MH150888 and MH150889.
